# Treatment-Specific Hippocampal Subfield Volume Changes With Antidepressant Medication or Cognitive-Behavior Therapy in Treatment-Naive Depression

**DOI:** 10.3389/fpsyt.2021.718539

**Published:** 2021-12-24

**Authors:** Hua-Hsin Tai, Jungho Cha, Faezeh Vedaei, Boadie W. Dunlop, W. Edward Craighead, Helen S. Mayberg, Ki Sueng Choi

**Affiliations:** ^1^Nash Family Center for Advanced Circuit Therapeutics, Icahn School of Medicine at Mount Sinai, New York, NY, United States; ^2^Thomas Jefferson University, Philadelphia, PA, United States; ^3^Department of Psychiatry and Behavioral Sciences, Emory University School of Medicine, Atlanta, GA, United States

**Keywords:** hippocampal atrophy, depression, antidepressant medication, cognitive behavioral theory, hippocampal tail

## Abstract

**Background:** Hippocampal atrophy has been consistently reported in major depressive disorder with more recent focus on subfields. However, literature on hippocampal volume changes after antidepressant treatment has been limited. The first-line treatments for depression include antidepressant medication (ADM) or cognitive-behavior therapy (CBT). To understand the differential effects of CBT and ADM on the hippocampus, we investigated the volume alterations of hippocampal subfields with treatment, outcome, and chronicity in treatment-naïve depression patients.

**Methods:** Treatment-naïve depressed patients from the PReDICT study were included in this analysis. A total of 172 patients who completed 12 weeks of randomized treatment with CBT (*n* = 45) or ADM (*n* = 127) were included for hippocampal subfield volume analysis. Forty healthy controls were also included for the baseline comparison. Freesurfer 6.0 was used to segment 26 hippocampal substructures and bilateral whole hippocampus from baseline and week 12 structural MRI scans. A generalized linear model with covariates of age and gender was used for group statistical tests. A linear mixed model for the repeated measures with covariates of age and gender was used to examine volumetric changes over time and the contributing effects of treatment type, outcome, and illness chronicity.

**Results:** Of the 172 patients, 85 achieved remission (63/127 ADM, 22/45 CBT). MDD patients showed smaller baseline volumes than healthy controls in CA1, CA3, CA4, parasubiculum, GC-ML-DG, Hippocampal Amygdala Transition Area (HATA), and fimbria. Over 12 weeks of treatment, further declines in the volumes of CA1, fimbria, subiculum, and HATA were observed regardless of treatment type or outcome. CBT remitters, but not ADM remitters, showed volume reduction in the right hippocampal tail. Unlike ADM remitters, ADM non-responders had a decline in volume in the bilateral hippocampal tails. Baseline volume of left presubiculum (regardless of treatment type) and right fimbria and HATA in CBT patients were correlated with a continuous measure of clinical improvement. Chronicity of depression had no effect on any measures of hippocampal subfield volumes.

**Conclusion:** Two first-line antidepressant treatments, CBT and ADM, have different effects on hippocampal tail after 12 weeks. This finding suggests that remission achieved via ADM may protect against progressive hippocampal atrophy by altering neuronal plasticity or supporting neurogenesis. Studies with multimodal neuroimaging, including functional and structural analysis, are needed to assess further the impact of two different antidepressant treatments on hippocampal subfields.

## Introduction

Major depressive disorder (MDD) affects more than 16.1 million American adults (6.7% of the US population) older than 18 each year (1) and is a significant public health concern throughout the world (2) First-line treatments for MDD include antidepressant medication (ADM) or cognitive-behavior therapy (CBT). Both treatments have roughly equivalent efficacy for achieving remission, which is the goal of acute-phase treatment for depression (3). Both treatments yield heterogeneous responses, with a large proportion of patients still having symptoms even after treatment. Several studies have reported that these variabilities in treatment responsiveness have been associated with specific clinical characteristics of MDD, such as the age of onset, comorbidity, duration of illness, past treatment, recurrence, or anxiety (4). Many studies, including the STAR^*^D trial, the largest controlled study of sequential MDD treatments, have investigated the effectiveness of different treatments for patients who fail to get sufficient relief from their initial ADM. Results of these studies indicate a declining probability of response with each additional treatment failure. However, the structural neurology underlying outcomes to treatments and their change with different types of treatment has received only little study to date.

The hippocampus (HC) is an important brain region involved in memory and emotion regulation. Smaller HC volumes have been consistently reported in MDD (5, 6). Despite well-documented whole HC atrophy in depression, there is mixed evidence concerning subregional morphology in HC volumes. Many studies show MDD patients have smaller volumes of HC subregions in the bilateral subiculum, Cornu Ammonis (CA)1, CA2, CA3, and tail regions (7, 8). In contrast, a recent study by Cao et al. did not find any difference in HC subfield volumes in MDD patients when compared to healthy controls (9). Additionally, MacQueen et al. examined the posterior HC volumes, reporting larger bilateral HC tail volumes at pre-treatment in eventual remitters than non-remitters treated with ADM (10). Following the MacQueen study, two recent studies with large cohorts replicated that larger HC tail volume is associated with remission achieved with ADM treatment (11, 12).

In the past decade, neuroimaging studies techniques have improved substantially, providing new insights into HC functional and structural brain alterations resulting from different treatments. Sheline et al. showed that ADM treatment counteracts the volume reduction in MDD patients (13). Additionally, other studies found that ADM stimulates neurogenesis in the dentate gyrus of adult rodents by increasing the number of neural progenitor cells (NPCs) (14, 15). A recent meta-analysis of treatment effects by Enneking et al. compared different modalities of effective treatments, including ADM, CBT, and electroconvulsive therapy (ECT) for MDD patients (16). ECT had the most robust volume changes in subcortical structures, including the hippocampus-amygdala complex, anterior cingulate cortex, and striatum. However, there was not sufficient evidence to determine the structural brain effects resulting from CBT treatment.

To date, there have been no studies directly comparing different subfield volume changes of the HC after two first-line treatments, CBT versus ADM. In order to understand treatment-specific effects on the HC, we analyzed longitudinal neuroimaging dataset collected at baseline and after 12 weeks of treatment in adults with MDD. We investigated differential changes patterns associated with (1) specific treatment (CBT versus ADM), (2) clinical outcome (remitter vs. nonremitter), and (3) chronicity (chronic vs. nonchronic).

## Methods

### Participants

MDD patients from the Predictors of Remission to Individual and Combined Treatments (PReDICT) study were included in this analysis (17). Briefly, in the PReDICT study adults aged 18–65 with moderate to severe, non-psychotic and treatment-naïve MDD were randomly assigned to 12 weeks of treatment with either CBT (16 1-h individual sessions) or one of two ADM, duloxetine (30–60 mg/day) or escitalopram (10–20 mg/day), in a 1:1:1 manner. Patients enrolled met DSM-IV criteria for MDD and had never previously received ≥4 weeks of ADM treatment or ≥4 sessions of an evidence-based psychotherapy for MDD or dysthymia. To ensure patients had at least moderate severity MDD, patients had to score ≥18 at screening and ≥15 at baseline on the 17-item Hamilton Depression Rating Scale (HDRS-17) (18). Patients were excluded if they met DSM-IV lifetime criteria for a psychotic disorder, bipolar disorder, or dementia, or if they had a current (past-year) diagnosis of eating disorder, dissociative disorder, or obsessive-compulsive disorder. Additionally, patients with any current primary DSM-IV disorder other than MDD were excluded (17). All participants provided written informed consent before beginning study procedures and the study was approved by the Emory Institutional Review Board.

A total of 172 patients (127 ADM and 45 CBT) completed the 12 weeks of treatment and had usable structural magnetic resonance imaging (MRI) scans at baseline and week 12. For comparison purposes, we analyzed MRI data from 40 healthy control subjects without a current or past history of psychiatric or neurological disorder who were scanned once as part of separate imaging studies conducted on the same scanner at Emory University.

### Clinical Outcomes and Chronicity

Patients were divided by their clinical outcomes and chronicity of their major depressive episode. Patients were split into four outcome groups based on the HDRS-17 change over the 12 weeks of treatment. Remitters had an HDRS-17 score ≤7 at both weeks 10 and 12. Responders without remission had a ≥50 % decrease from baseline but did not meet the remission definition. Partial responders had a 30–49% decrease in HDRS-17 score. Non-responders had a <30% decrease. Additionally, patients were divided into two groups (chronic and non-chronic) using a cut-point of 104 weeks (i.e., 2 continuous years) for their major depressive episode length at the time of screening. Patients were also subdivided by the reported number of depressive episodes (1, 2, and ≥3).

### MRI Acquisition

MRI scanning was performed using a 3T Siemens TIM Trio (Siemens Medical Systems, Erlangen, Germany) at Emory University. Two longitudinal high-resolution T1-weighted magnetization-prepared rapid gradient echo (MPRAGE) scans were acquired at the start of the study (baseline) and after 12 weeks of treatment. The acquisition parameters are the following: sagittal slice orientation; slice thickness = 1.0 mm; in-plane resolution = 1.0 × 1.0 mm; matrix = 240 × 240; repetition time = 2,300 ms; inversion time = 900 ms; flip angle = 9°.

### Hippocampal Subfield Volume Calculation

The hippocampal subfield segmentation module implemented in Freesurfer 6.0 was used to estimate a total of 26 different segmented subfields, 13 for each side of the brain (19). A further description of the algorithm can be found in Iglesias et al. (20). The segmented hippocampal subfield masks were carefully checked visually, and no substantial error was detected. The volumes of the hippocampal subfield were extracted based on the segmented masks. In addition, the whole HC volume of each hemisphere was also extracted. The volumes in all discussed analyses use the following normalization formula to account for different total brain volumes of subregions between subjects (**Equation 1**). The estimation of total intracranial volume of each subject was calculated using the standard Freesurfer pipeline. Further discussion of subfield volumes in this paper will assume the use of the normalized volume unless otherwise noted.

**Equation 1**. Brain Volume Normalization:


$$\begin{array}{l} \%Normalized\;Volume\;of\;Interest\\ \;=\;\frac{Hippocampal\;Subfield}{Total\;Intracranail\;Volume}\;*\;100 \end{array}$$

### Statistical Analysis

For the current analyses, the two antidepressants were combined to form one ADM group. This was done to increase statistical power and is justified based on the absence of any literature demonstrating differences in clinical efficacy or neurological volume effects between these medications (Supplementary Table 1). All statistical analyses were conducted using Jamovi software (21). A general linear model was used to compare the group difference at baseline including: (1) healthy controls vs. treatment-naive MDD, and (2) CBT-treated vs. ADM-treated remitters. A linear mixed model with repeated measures was used to examine the longitudinal volume changes of the HC subfield regardless of treatment, outcome, or chronicity. Furthermore, a linear mixed model with interaction evaluated: (1) treatment-specific effect—time × treatment (CBT-treated vs. ADM-treated remitters); (2) treatment-specific outcome effect—time × outcome (remitters vs. non-responders); and (3) chronicity effect—time × chronicity (chronic vs. nonchronic). Following any significant interaction findings, a *post-hoc* paired *t-*test was performed to validate the results. Lastly, a correlation analysis was performed between HC subfield volume at baseline and volume changes (**Equation 2**) over time and HDRS-17 changes (**Equation 3**). All statistical analyses included age and gender as covariates. Two statistical significance thresholds were applied to all analyses: (1) p_Uncorrected_ <0.05 and (2) p_Bonferroni_ <0.05, which takes 26 different regions (considering both left and right brain), to get p_Uncorrected_ <0.0019. Both alphas are reported to capture potentially significant factors.

**Equation 2**. Brain Volume Change:


$$\begin{array}{l} \text{Δ}BV=\;BV_{Baseline}-BV_{Wk12}\; \end{array}$$

where *BV*_*Baseline*_ is the %Normalized Volume of Interest at baseline and *BV*_*Wk*12_ is the % Normalized Volume of Interest at week 12.

**Equation 3**. HDRS-17 Change:


$$\begin{array}{l} \text{Δ}HDRS=\;\frac{HDRS_{Baseline1}-HDRS_{Wk2}}{HDRS_{Baseline}}\;*\;100 \end{array}$$

where *HDRS*_*Baseline*_ is the HDRS-17 score at baseline and *HDRS*_*Wk*12_ is the HDRS-17 score at week 12.

**Figure 1:**
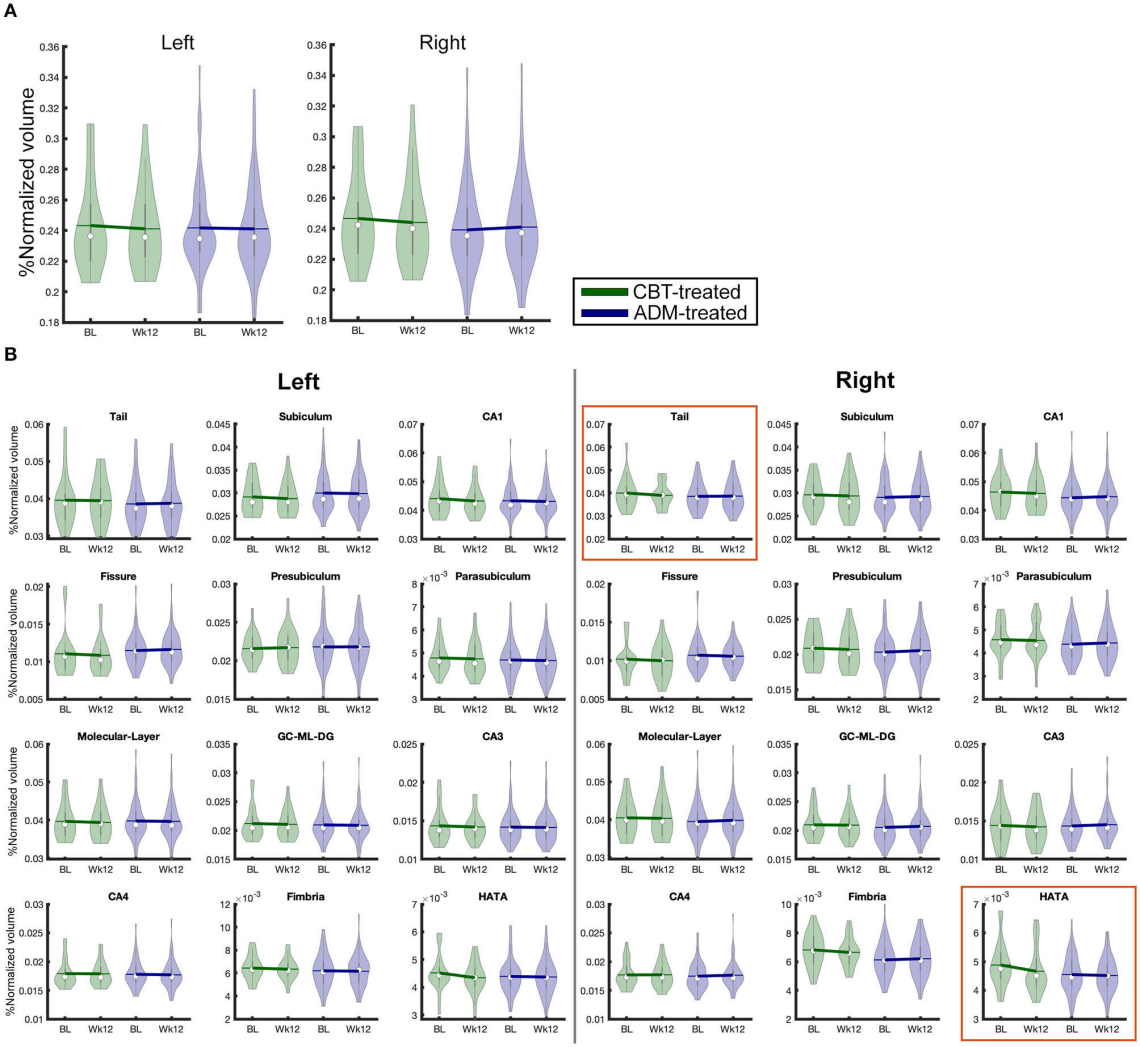
Total volumes of 26 hippocampal subfield used in analysis broken by treatment group and time. BL, Baseline; Wk12, Week 12; CBT, cognitive behavioral therapy; ADM, antidepressant medications. **(A)** whole hippocampus, **(B)** hippocampal subfields.

A legend for the 26 different subfields (left and right) is as follows (Figure 1): (1) hippocampal tail, (2) subiculum, (3) CA1, (4) hippocampal fissure, (5) presubiculum, (6) parasubiculum, (7) molecular layer, (8) Granule Cell Molecular Layer of the Dentate Gyrus (GC-ML-DG), (9) CA3, (10) CA4, (11) fimbria, (12) Hippocampal Amygdala Transition Area (HATA), and (13) unilateral whole hippocampus.

## Results

### Clinical Characteristics and Treatment Outcomes

The demographic and clinical characteristics of the participants are presented in Table 1. There was no significant difference in age between the healthy controls (36.5 ± 8.57 years) and the MDD group (39 ± 11.5 years). There were also no significant differences in age among the four treatment outcome groups. At the baseline, MDD patients had an HDRS-17 score of 18.9 ± 3.32. After 12 weeks treatment, MDD patients had a HDRS-17 score of 7.22 ± 5.72, which was significantly reduced (t (171) = 24.7, *p* < 0.001) and 85 patients (49.4%) had remitted. Detailed HC subfield volumes are presented in Supplementary Table 2.

**Table 1:** Demographics and baseline characteristics of participants with MDD.

**Characteristic**	**Study group**	
	**Healthy controls**	**MDD**
N	40	172
Female	24 (60.0)	104 (60.5)
Age, mean (SD), yrs^A^	36.5 (8.76)	39.0 (11.5)
Outcome, treatment N		
Remitter, total/ADM/CBT^A^	-	85 / 63 / 22
Responder, total/ADM/CBT^A^	-	36 / 29 / 7
Partial-responder, total/ADM/CBT^A^	-	25 / 17 / 8
Non-responder, total/ADM/CBT^A^	-	26 / 18 / 8
No. lifetime episodes		
1	-	90 (52.3)
2	-	26 (15.1)
≥3	-	56 (32.6)
Chronic episode (≥2 yrs)	-	57 (33.1)
**Symptom severity, HDRS-17 score**	**Baseline MDD**	**Week 12 MDD**
All participants, mean (SD)^A^	18.9 (3.32)	7.22 (5.72)
Remitter, mean (SD)^A^	18.7 (3.51)	2.82 (2.21)
Responder, mean (SD)^A^	20.2 (3.11)	7.64 (2.31)
Partial-responder, mean (SD)^A^	18.3 (2.43)	11.7 (1.95)
Non-responder, mean (SD)^A^	18.6 (3.45)	16.7 (4.12)

*MDD, Major depressive disorder; HDRS-17, Hamilton Depression Rating Scale; ADM, Antidepressant medications; CBT, Cognitive behavioral therapy*.

A *Unless otherwise indicated, data are expressed as number (percentage) of participants*.

#### Baseline Hippocampal Subfield Volume Differences Between Healthy Controls and Treatment Naive MDD Patients

**Figure 2:**
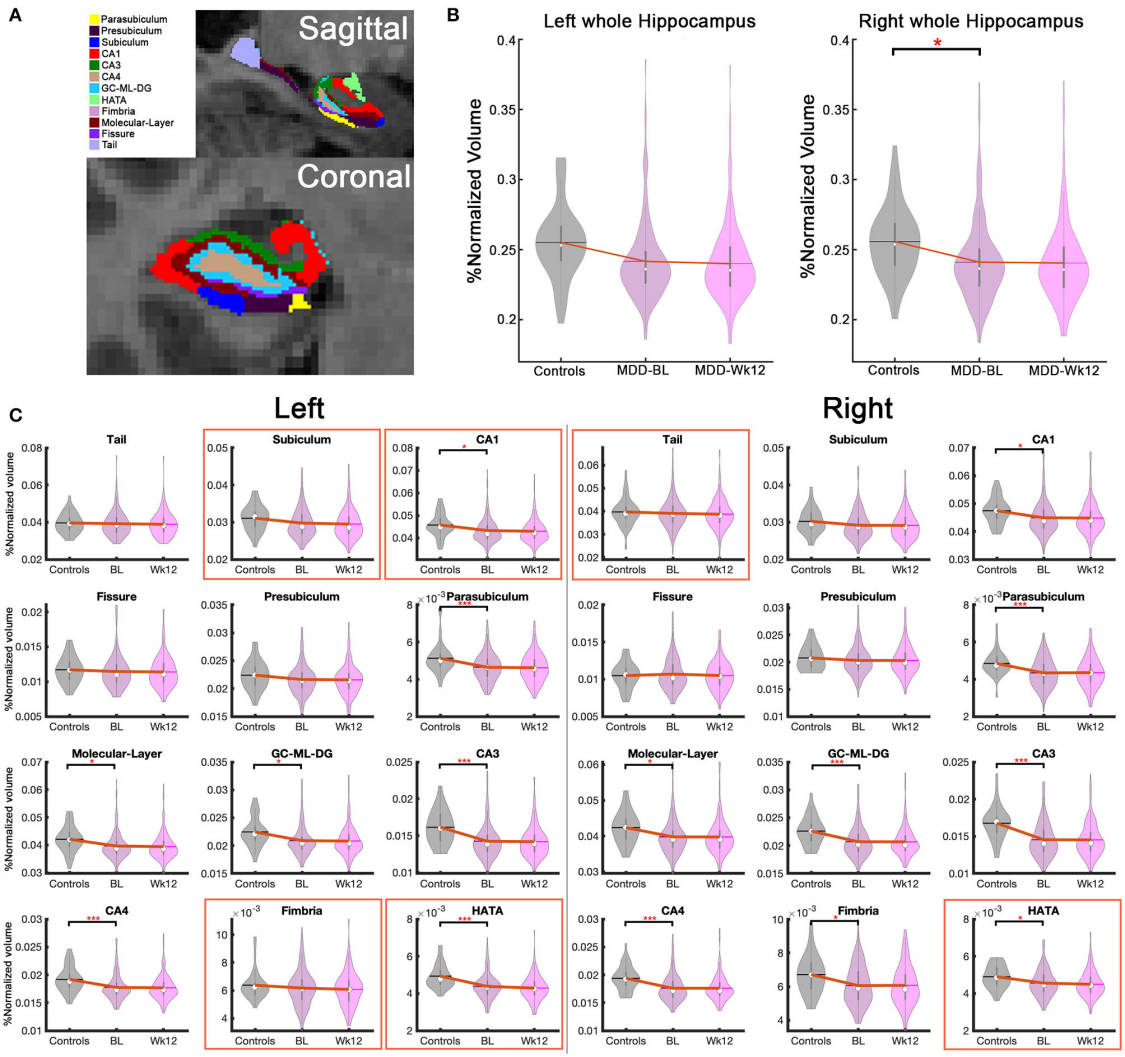
Hippocampal subfield volume differences between treatment-naive MDD and Healthy controls, and hippocampal volume changes over 12 weeks of treatments. **(A)** Hippocampal subfield volumes in coronal and sagittal images. **(B)** Bilateral whole hippocampus volume changes. Right whole hippocampus volume at baseline was significantly smaller than healthy controls (p_Uncorrected_ < 0.05) **(C)** 26 hippocampal subfield volume in healthy controls, baseline and week 12 in treatment-naive MDD patients. Fifteen substructures including bilateral CA1, CA3, CA4, parasubiculum, GC-ML-DG, molecular layer, HATA, and right fimbria are shown significant volume reduction in the MDD patients compared to healthy controls (p_Uncorrected_ < 0.05). The bilateral CA3, bilateral CA4, bilateral Parasubiculum, left HATA, and right GC-ML-DG results survived multiple comparison corrections (p_Bonferroni_ < 0.05). Notably, six subregions (red box), including bilateral HATA, left subiculum, left CA1, left Fimbria, and right tail, showed a volume reduction between baseline and week 12 (p_Uncorrected_ < 0.05). The left HATA is highly significant (p_Bonferroni_ <0.05). *p_Uncorrected_ < 0.05, ***p_Bonferroni_ < 0.05, BL, baseline; Wk12, week 12.

Fifteen hippocampal subfields out of 26 regions were significantly smaller in MDD patients compared to healthy controls (p_Uncorrected_ <0.05) at baseline: (1–2) bilateral CA1, (3–4) bilateral CA3, (5–6) bilateral CA4, (7–8) bilateral parasubiculum, (9–10) bilateral GC-ML-DG, (11–12) bilateral Molecular layer, (13–14) bilateral HATA, (15) right Fimbria (Supplementary Table 3). Interestingly, the bilateral whole HC volumes were also smaller in MDD patients although this finding did not survive after Bonferroni correction (Supplemantary Table 3). With Bonferroni multiple comparison correction (p_Bonferroni_ <0.05), only eight subregions out of 15 significant substractures survived including bilateral CA3, CA4, parasubiculum, left HATA, and right GC-ML-DG (Figure 2).

#### Baseline Hippocampal Subfield Volume Differences Between ADM- and CBT-Treated Patients

**Figure 3:**
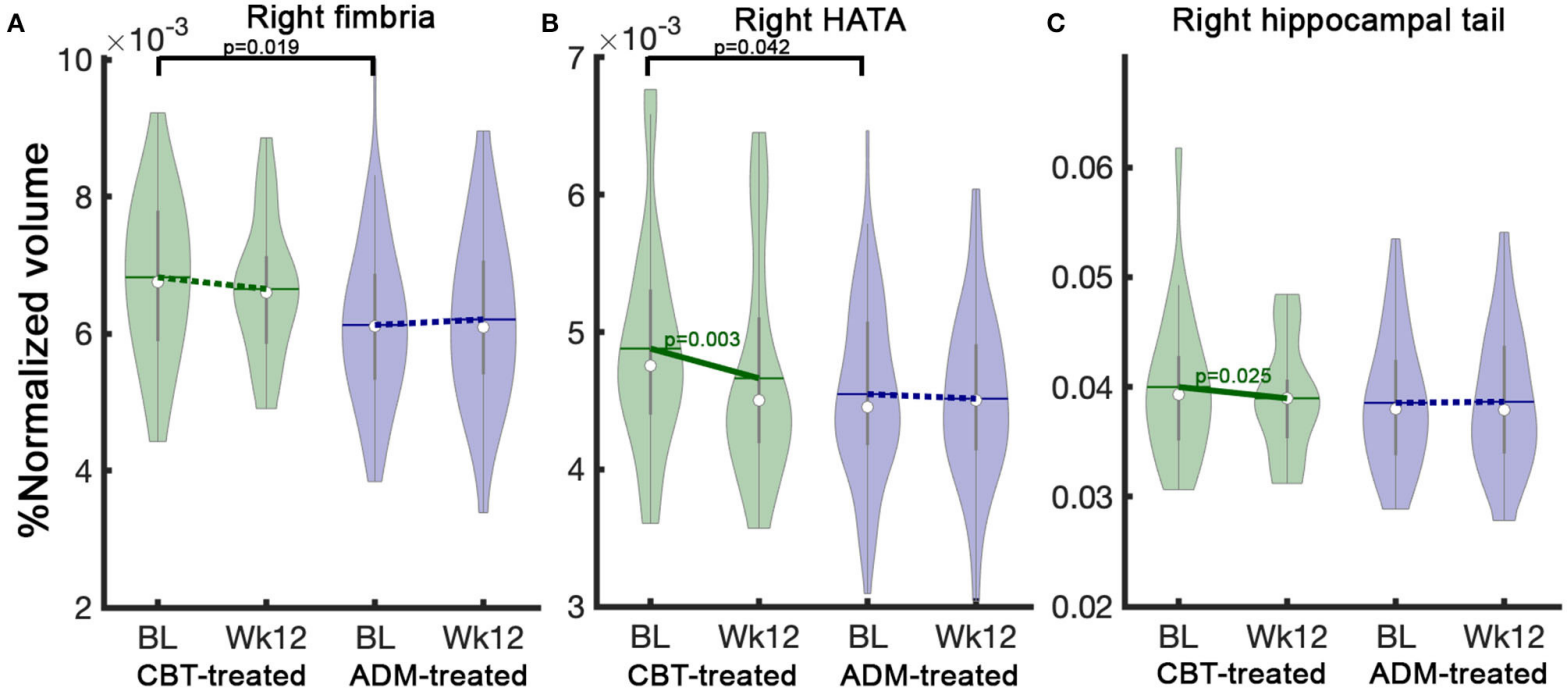
Treatment specific hippocampal volume differences between CBT-treated and ADM-treated remitters at baseline and changes after 12 weeks of treatment. CBT-treated remitters showed larger HC volumes at baseline in **(A)** Right fimbria and **(B)** Right HATA. Notably, CBT-treated patients show significant volume reductions over 12 weeks of treatment in **(B)** Right HATA and **(C)** Right hippocampal tail. In contrast, ADM-treated patients showed no changes. The solid line represents *p*-values from the *post-hoc* paired *t*-test between baseline and week 12 volume. BL, baseline, Wk12, week 12. CBT, cognitive behavioral therapy; ADM, antidepressant medication.

There were no statistical baseline differences in hippocampal subfield volumes between ADM-treated (*n* = 127) and CBT-treated (n = 45) patients. However, ADM-treated remitters (*n* = 63) showed smaller hippocampal baseline volumes in right fimbria (p_Uncorrected_ = 0.019) and HATA (p_Uncorrected_ = 0.042) than CBT-treated remitters (*n* = 22) (Figures 3A,B).

#### Baseline Hippocampal Subfield Volume Associated With Outcome

Left presubiculum volume at baseline showed a significant relationship with the percentage change of HDRS-17 scores. Larger HC volume in the left presubiculum was positively associated with clinical improvement (R^2^ = 0.056, *p* = 0.038). In addition, the CBT-treated group (*n* = 45) showed a positive relationship between clinical improvement and volume of the right fimbria (R^2^ = 0.182, *p* = 0.022) and HATA (R^2^ = 0.169, *p* = 0.032), but no such relationship was obtained for the ADM-treated patients (*n* = 127) (Figure 1). *Post-hoc* analysis by group found that remitters had larger volumes at baseline in the left presubiculum (p_Uncorrected_ = 0.019) and parasubiculum (p_Uncorrected_ = 0.04) than non-responders regardless of treatment. Within ADM-treated patients, there were no volumes that showed statistically significant differences between remitters and non-responders, but within CBT-treated patients, remitters had larger baseline volumes than non-responders in the whole right HC (p_Uncorrected_ = 0.025) and various subregions including left GC-ML-DG (p_Uncorrected_ = 0.032) and CA4 (p_Uncorrected_ = 0.04), and right CA1 (p_Uncorrected_ = 0.011), GC-ML-DG (p_Uncorrected_ = 0.02), molecular layer (p_Uncorrected_ = 0.029), HATA (p_Uncorrected_ = 0.029). Additional statistical results of these comparisons are presented in Supplementary Table 4.

#### Baseline Hippocampal Subfield Volume Associated With Chronicity, Number of Episodes, or Symptom Severity

There were no statistical differences in any HC subfields between chronic and non-chronic groups or by number of past episodes at baseline. Additionally, there were no interaction effects of time and chronicity (Supplementary Table 5). Lastly, there was no significant findings between symptom severity (HAMD score) and baseline volumes.

#### Hippocampal Subfield Volume Changes Across Treatments (Time Effects)

Regardless of treatment (ADM or CBT), MDD patients demonstrated longitudinal HC volume reduction over 12 weeks in six subregions, including bilateral HATA, left subiculum, left CA1, left Fimbria, and right tail (p_Uncorrected_ < 0.05). Notably, bilateral HATA and left CA1 were smaller compared to healthy controls at baseline and continued to decrease over 12 weeks with treatment (Figure 2). Only the left HATA survived after multiple comparison corrections (p_Bonferroni_ < 0.05). The left whole HC volume also decreased over time (p_Uncorrected_ < 0.05). Figure 2 presents HC volume changes over 12 weeks of treatment and Supplementary Table 4 presents detailed statistical results.

#### Treatment-Specific Hippocampal Subfield Volume Changes (Treatment Effects)

Only remitters are included in treatment-specific change analyses because finding pathological changes in the remitters could help explain the clinical outcomes as they showed the largest improvement. Interestingly, there were differential treatment-specific HC subfield volume changes in the right HC tail (p_Uncorrected_ = 0.035) and HATA (p_Uncorrected_ = 0.03): remitters to CBT demonstrated decreased volumes over time, whereas ADM remitters showed no volume changes (Supplementary Table 6). Figure 3 presents treatment-specific volume changes in the right HC tail and HATA.

#### Hippocampal Subfield Volume Changes Associated With Outcome (Outcome Effects)

**Figure 4:**
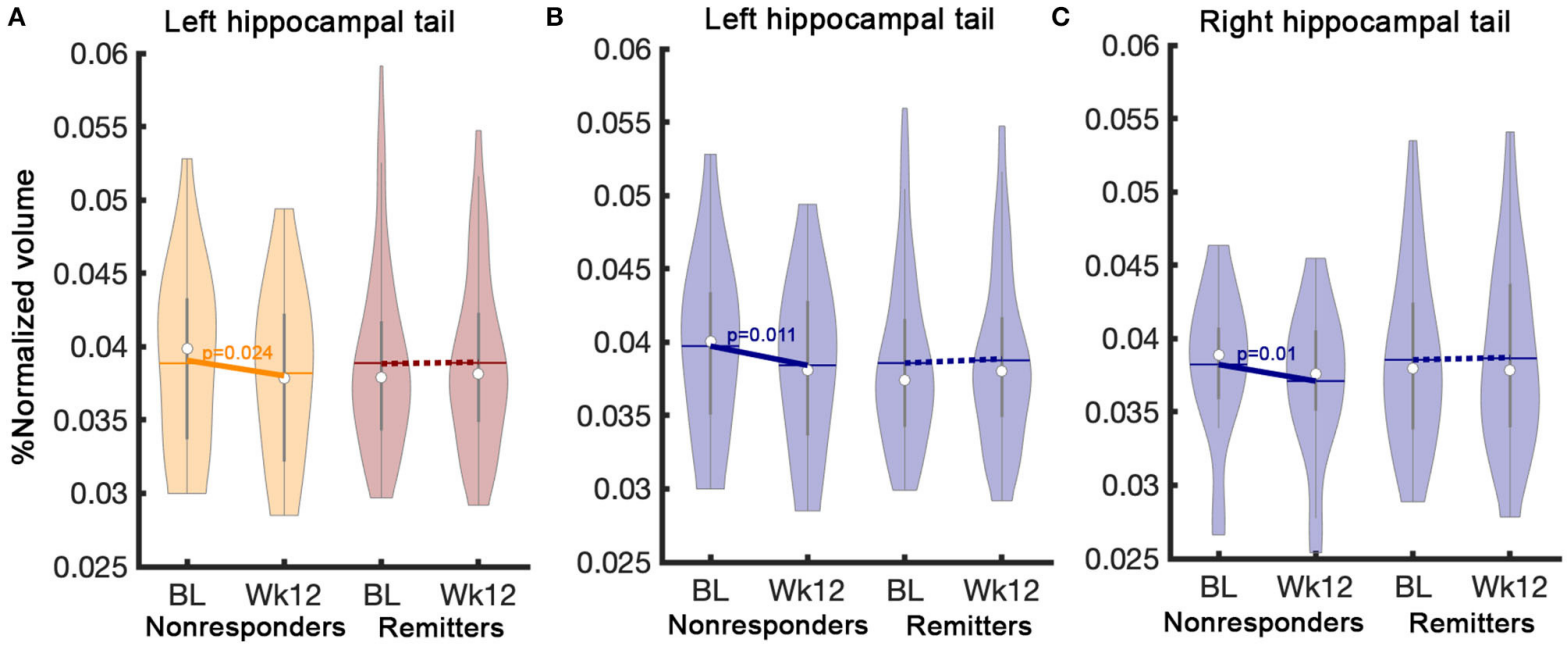
Interaction effects between time and outcome (remitters vs. non-responders). A significant interaction effect between remitters (*n* = 85) and non-responders (*n* = 26) regardless of treatment was found **(A)** in the left hippocampal tail. *Post-hoc* analysis in the ADM-treated patients show differential volume changes between remitters (*n* = 63) and non-responder (*n* = 18) **(B)** in the left and **(C)** right hippocampal tails. The solid line represents *p*-values from the *post-hoc* paired *t*-test between baseline and week 12 volume. BL, Baseline; Wk12, Week 12; CBT, cognitive behavioral therapy; ADM, antidepressant medication.

There was no statistical relationship between clinical improvement (percent change of HDRS-17 scores) and HC subfield volume changes. In addition, there were no significant interaction effects in time-by-outcome among four different outcome groups, including remitters, responders, partial responders, and non-responders. However, the left HC tail (p_Uncorrected_ = 0.03) had a significant interaction effect between remitters and non-responders regardless of treatment. Left HC tail volume in non-responders significantly decreased over time, but no volume change occurred in remitters (Supplementary Table 7). *Post-hoc* analysis in each treatment group revealed that the left tail volume reduction was only significant in the ADM-treated patients. There were no significant interaction effects between time and outcomes in the CBT group. Interestingly, the *post-hoc* analysis also found volume reduction in the right HC tail (F = 6.37, p_Uncorrected_ = 0.01) with the ADM-treated patients (Figure 4).

## Discussion

The main aim of this study was to explore the differential effects of treatment types, treatment outcomes, and chronicity of depression on hippocampal substructures using a longitudinal neuroimaging dataset from baseline and following 12 weeks of treatments. This study is the first to directly compare the effects of antidepressant medication vs. cognitive behavior therapy in hippocampal subfield volume changes in a large cohort of treatment-naive MDD (*n* = 172) patients. Differential treatment-specific volume changes were found in HC subregions between remitters to ADM and CBT. CBT remitters showed a volume reduction in the right hippocampal tail, which did not occur in ADM-treated remitters. In contrast, non-responders in ADM-treated patients showed volume reductions in the bilateral hippocampal tails while remitters in ADM-treated patients had unchanged volumes. This finding suggests the importance of hippocampal tail volumes in considering the effects of ADM treatment, consistent with prior research (10). ADM may protect against progressive hippocampal atrophy by altering neuronal plasticity or supporting neurogenesis (22). The other notable results from this study are: (1) various hippocampal subregion volumes are smaller in patients with MDD compared to healthy controls, which is consistent with previous findings (6); (2) six hippocampal subregion volumes demonstrated decreases after 12 weeks of treatment regardless of treatment or outcome, particularly the highly significant finding of left HATA volume reduction; (3) larger baseline right fimbria and HATA volumes in the CBT-treated patients were positively associated with clinical improvement.

Consistent with previous reports, the MDD patients in our study showed significantly smaller bilateral whole HC volumes at baseline compared to healthy controls (5). Additionally, our results indicate various HC subregions are smaller in MDD compared to healthy controls. Roddy et al. recently suggested that hippocampal subregion atrophy starts with principal substructures, including CA regions, and possibly progresses to an expanding pattern of substructure involvement including subiculum, dentate, and molecular layer. Furthermore, they reported no peripheral HC substructure involvement, including HATA, tail, presubiculum, and parasubiculum. However, our results show significant volume reductions in both principal and peripheral regions in treatment-naive depression and no relation of these changes to chronicity of depressive episode.

Two recent studies reported the importance of hippocampal tail volumes in the prediction of remission with antidepressant medication (11, 12). Maller et al. showed that MDD patients had larger hippocampal tail volumes than healthy controls at baseline, and this larger volume was associated with both a diagnosis of MDD and a greater chance of remission to ADM (11). In contrast, Nogovitsyn et al. reported smaller hippocampal tail volumes in MDD patients than healthy controls, and the size of hippocampal tail volumes in depression patients was positively associated with remission status, including early and later remission with antidepressant medication. Although the two studies reported opposite results in baseline hippocampal tail volumes in MDD vs. control subjects, both studies agreed that larger hippocampal volume was associated with remission in ADM-treated patients. Our analysis did not show a difference in hippocampal tail size compared to healthy controls at baseline. Furthermore, there were no differences between remitters and non-responders in hippocampal tails at baseline. However, these two studies also showed that the HC tail did not change over time in ADM remitters suggesting that hippocampal tail volumes were sensitive to ADM treatment. Additionally, previous studies demonstrated HC volume increase with 8 weeks of citalopram treatment as well as with 3 years of treatment with various antidepressant medications (23, 24). These findings may reflect the effects of ADM on suppressing toxic stress and enhancing neurogenesis and synaptic plasticity as previously reported (25–28).

We found that the right fimbria and HATA volumes at baseline were positively associated with clinical improvement specifically in CBT-treated patients. The fimbria is an important white matter relay connecting the HC to the paraventricular nucleus of hypothalamus and other limbic regions. A previous study demonstrated that deep brain stimulation of fimbria-fornix enhanced learning and memory capability (29). Additionally, HATA atrophy might affect the integrity of the hippocampal-amygdala network that is critical to cognition. This suggests that patients with significant decline in fimbria and HATA volume are less responsive to CBT and, therefore, may warrants further research in MDD patients. Nevertheless, the benefits achieved with CBT treatment of MDD is likely related to other neural effects rather than hippocampal tail volume changes.

Hippocampal atrophy with chronicity has been consistently reported in depression. Previously Sheline et al. reported that longer untreated periods in recurrent depression are associated with volume decline in the HC (14). Unlike their report, we found no baseline differences between chronic and non-chronic groups and no significant differences in volumes changes during treatment between these subgroups. This discrepancy may be due to reduced statistical power stemming from group comparisons using a two year cut off instead of a correlation analysis employing a continuous measure of episode duration. We used group analysis because retrospective self-report for duration of illness is often highly inaccurate, so categorical classification is a more conservative approach to the data (30). Our analysis also showed no statistical relationship between depression severity and volume reduction at baseline as previously reported by Frodl et al. (31) and Nils et al. (32). Furthermore, we found no significant relationship between baseline severity and volume changes after 12 weeks of treatment (Supplementary Table 8).

Interestingly, after 12-weeks of antidepressant treatment, six HC substructures show significant longitudinal volume reduction regardless of treatment and outcomes. Left HATA and left CA1 were smaller at baseline compared to healthy controls, and they continued to decrease over 12 weeks of treatment. In contrast, the remainder of the abnormal substructures at baseline compared to healthy controls show no longitudinal changes. This finding is consistent with our chronicity findings which show no differences between chronic and non-chronic groups. These findings suggest there is no progressive atrophy in these HC substructures once atrophy happens in depressed patients, which may suggest a floor effect in HC subfields.

A recent study using the same imaging dataset, but analyzing functional magnetic resonance imaging (fMRI), in treatment-naive depressed patients treated with ADM or CBT identified that the functional connectivity of the subcallosal cingulate cortex (SCC) with three brain regions (ventromedial frontal, ventrolateral frontal/anterior insula, and dorsal midbrain) differed between remitters to CBT or ADM (5). Greater hyperconnectivity between the SCC, one of the critical hub regions in depression networks, and these three regions predicted remission with ADM; in contrast, hypo-connectivity predicted remission with CBT (5). Further studies may consider volumetric analysis. Combining the multiple structural data with volumetric-connectivity associations could create a more multifactorial approach for better understanding MDD. Additionally, direct comparison with recurrent or treatment-resistant depression cohorts could also be considered to evaluate the effects of past treatments and disease progression toward treatment-resistant depression.

Our study has limitations, including different sample sizes between ADM- and CBT-treated patients and HC subfield segmentation with a 1mm resolution using a single modality. There were more remitters in the ADM group (*n* = 63) than CBT (*n* = 22) due to two different antidepressant medications (duloxetine and escitalopram) in our protocol. Additionally, the sample size of CBT non-responders (*n* = 8) is relatively small, limiting the statistical inferences that can be drawn regarding outcome effects. In addition to the sample size, the recent version of Freesurfer (Freesurfer 6.0) allows both T1 and T2 images to improve HC subfield segmentation performance. However, our analysis only used T1 images because there were no reliable T2 images available. Importantly, interaction effects, including treatment- and outcome-specific findings, did not survive after Bonferroni multiple comparison corrections. The small magnitude of changes in HC subfield volume limited our ability to find significance after multiple comparison corrections.

To the best of our knowledge, there have been no direct comparisons of hippocampal subfield morphological changes between antidepressant medication and cognitive behavior therapy with treatment outcomes in a large sample. This study advances our understanding of first-line antidepressant treatment effects on hippocampal substructures. In particular, we demonstrate differential treatment-specific effects on hippocampal tail volume changes after 12 weeks of treatments. Remitters with antidepressant medication had preserved hippocampal tail volume but CBT remitters did not. We further show hippocampal tail volume reduction in non-responders with antidepressant medication. The observation that no hippocampal tail volume changes in remitters with antidepressant medication may reflect the action of suppressing stress toxic effects and increasing neurogenesis factors, consistent with animal studies (33).

## Data Availability Statement

The data that support the findings of this study are available from the corresponding author, upon reasonable request.

## Ethics Statement

The studies involving human participants were reviewed and approved by the Emory Institutional Review Board and the Grady Hospital Research Oversight Committee. The patients/participants provided their written informed consent to participate in this study.

## Author Contributions

H-HT, JC, and KC: neuroimaging analysis, statistical analysis, and co-writing paper. FV: neuroimaging analysis. BD: co-writing protocols, inclusion/recruitment, clinical assessment, and co-writing paper. WC: co-writing protocols, grant recipient, inclusion/recruitment, and clinical assessment. HM: co-writing protocols, grant recipient (principal investigator), and co-writing paper. All authors contributed to the article and approved the submitted version.

## Funding

This work was supported by the National Institutes of Health (HM, P50 MH077083; WC, RO1MH080880; and David S. Stephens, UL1 RR025008, M01 RR0039).

## Conflict of Interest

BD has received research support from Acadia, Compass, Aptinyx, NIMH, Sage, and Takeda, and has served as a consultant to Greenwich Biosciences, Myriad Neuroscience, Otsuka, Sage, and Sophren Therapeutics. The remaining authors declare that the research was conducted in the absence of any commercial or financial relationships that could be construed as a potential conflict of interest.

## Publisher's Note

All claims expressed in this article are solely those of the authors and do not necessarily represent those of their affiliated organizations, or those of the publisher, the editors and the reviewers. Any product that may be evaluated in this article, or claim that may be made by its manufacturer, is not guaranteed or endorsed by the publisher.

## Supplementary Material

Supplementary Table 1 Hippocampal subfield volume change associated with different medication treatment. Cornu Ammonis (CA), Granule Cell Molecular Layer of the Dentate Gyrus (GC-ML-DG), Hippocampal Amygdala Transition Area (HATA).

Supplementary Table 2 Volumes are given as mean ± SD in cubic millimeters (mm^3^); eTIV, estimated total intracranial volume; Cornu Ammonis (CA), Granule Cell Molecular Layer of the Dentate Gyrus (GC-ML-DG), Hippocampal Amygdala Transition Area (HATA).

Supplementary Table 3 Baseline effect between healthy controls and Baseline MDD. Cornu Ammonis (CA), Granule Cell Molecular Layer of the Dentate Gyrus (GC-ML-DG), Hippocampal Amygdala Transition Area (HATA).

Supplementary Table 4 Baseline effect between Baseline and Week 12. Cornu Ammonis (CA), Granule Cell Molecular Layer of the Dentate Gyrus (GC-ML-DG), Hippocampal Amygdala Transition Area (HATA).

Supplementary Table 5 Chronicity effect between Baseline and Week 12. Cornu Ammonis (CA), Granule Cell Molecular Layer of the Dentate Gyrus (GC-ML-DG), Hippocampal Amygdala Transition Area (HATA).

Supplementary Table 6 Treatment effect between Baseline and Week 12. Cornu Ammonis (CA), Granule Cell Molecular Layer of the Dentate Gyrus (GC-ML-DG), Hippocampal Amygdala Transition Area (HATA).

Supplementary Table 6a Baseline differences of different treatments. Cornu Ammonis (CA), Granule Cell Molecular Layer of the Dentate Gyrus (GC-ML-DG), Hippocampal Amygdala Transition Area (HATA).

Supplementary Table 7 Outcome effect between Baseline and Week 12. Cornu Ammonis (CA) Granule Cell Molecular Layer of the Dentate Gyrus (GC-ML-DG), Hippocampal Amygdala Transition Area (HATA).

Supplementary Table 8 HDRS score changes with volume change. Cornu Ammonis (CA), Granule Cell Molecular Layer of the Dentate Gyrus (GC-ML-DG), Hippocampal Amygdala Transition Area (HATA), Hamilton Depression Rating Scale (HDRS).
