# Supplementary material for: Treatment-Specific Hippocampal Subfield Volume Changes With Antidepressant Medication or Cognitive-Behavior Therapy in Treatment-Naive Depression
Source: Front Psychiatry. 2021 Dec 24;12:718539. doi: 10.3389/fpsyt.2021.718539 (PMC8739262; doi:10.3389/fpsyt.2021.718539)
Supplement: Supplementary Table 1 — Hippocampal subfield volume change associated with different medication treatment. Cornu Ammonis (CA), Granule Cell Molecular Layer of the Dentate Gyrus (GC-ML-DG), Hippocampal Amygdala Transition Area (HATA). [file Table_1.pdf]

Table 1. Hippocampal subfield volume change associated with different medication treatment

	All	
**Left Hippocampus**	F	p
Tail	0.603	0.439
Subiculum	4.770E-04	0.983
CA1	8.740E-04	0.976
Fissure	0.144	0.705
Presubiculum	0.225	0.636
Parasubiculum	1.519	0.220
Molecular layer	0.022	0.884
GC-ML-DG	0.0131	0.909
CA3	0.143	0.706
CA4	0.002	0.963
Fimbria	0.011	0.917
HATA	0.0079	0.929
Whole	0.0338	0.854
**Right Hippocampus**		
Tail	3.101	0.081
Subiculum	0.279	0.598
CA1	0.257	0.613
Fissure	0.258	0.612
Presubiculum	0.002	0.962
Parasubiculum	0.015	0.904
Molecular layer	0.013	0.911
GC-ML-DG	1.050E-04	0.992
CA3	0.145	0.704
CA4	0.004	0.951
Fimbria	0.198	0.657
HATA	0.242	0.624
Whole	0.254	0.615
