# Supplementary material for: Treatment-Specific Hippocampal Subfield Volume Changes With Antidepressant Medication or Cognitive-Behavior Therapy in Treatment-Naive Depression
Source: Front Psychiatry. 2021 Dec 24;12:718539. doi: 10.3389/fpsyt.2021.718539 (PMC8739262; doi:10.3389/fpsyt.2021.718539)
Supplement: Supplementary Table 2 — Volumes are given as mean ± SD in cubic millimeters (mm3); eTIV, estimated total intracranial volume; Cornu Ammonis (CA), Granule Cell Molecular Layer of the Dentate Gyrus (GC-ML-DG), Hippocampal Amygdala Transition Area (HATA). [file Table_2.pdf]

Table 2. Hippocampal Subfield Volumes (mm^3)

	Controls	MDD Baseline	MDD Week12
eTIV	1379154 ± 171460	1410820 ± 183677	1413807 ± 180717
**Left Hippocampus**			
Tail	541 ± 59.7	545 ± 67.5	543 ± 67.2
Subiculum	424 ± 34.3	416 ± 49.6	413 ± 50.9
CA1	625 ± 60	605 ± 68.4	603 ± 68.4
Fissure	160 ± 25.4	159 ± 26.5	159 ± 25.6
Presubiculum	306 ± 28.3	302 ± 36	302 ± 38
Parasubiculum	70 ± 9.94	65 ± 10.9	64.9 ± 11.2
Molecular layer	575 ± 48.7	553 ± 53.1	552 ± 54.8
GC-ML-DG	306 ± 27.3	291 ± 29.6	291 ± 29.1
CA3	220 ± 26.3	198 ± 26.1	198 ± 25.3
CA4	262 ± 26.3	247 ± 25.6	247 ± 25.1
Fimbria	87.1 ± 12.3	86.5 ± 19.7	85.4 ± 18.2
HATA	67.7 ± 9.81	61.3 ± 9.52	60.1 ± 8.94
Whole	3484 ± 274	3371 ± 312	3360 ± 318
**Right Hippocampus**			
Tail	544 ± 82.6	544 ± 70.2	541 ± 68.4
Subiculum	413 ± 32.2	408 ± 48.7	408 ± 48.3
CA1	650 ± 66.2	628 ± 78.9	628 ± 77.5
Fissure	143 ± 19.3	149 ± 26.3	147 ± 24.2
Presubiculum	284 ± 38.3	284 ± 34.1	284 ± 34
Parasubiculum	66.6 ± 10.1	60.5 ± 9.48	60.9 ± 10.3
Molecular layer	579 ± 51.1	556 ± 60.3	557 ± 58.2
GC-ML-DG	309 ± 27.6	289 ± 33	289 ± 30.7
CA3	229 ± 26.4	203 ± 30.3	203 ± 28.9
CA4	265 ± 23.8	246 ± 28	246 ± 26.3
Fimbria	92 ± 15.9	84.8 ± 16.9	85.4 ± 17.9
HATA	67 ± 8.2	63.7 ± 9.63	63 ± 9.16
Whole	3498 ± 307	3366 ± 343	3366 ± 331
