# Supplementary material for: Treatment-Specific Hippocampal Subfield Volume Changes With Antidepressant Medication or Cognitive-Behavior Therapy in Treatment-Naive Depression
Source: Front Psychiatry. 2021 Dec 24;12:718539. doi: 10.3389/fpsyt.2021.718539 (PMC8739262; doi:10.3389/fpsyt.2021.718539)
Supplement: Supplementary Table 3 — Baseline effect between healthy controls and Baseline MDD. Cornu Ammonis (CA), Granule Cell Molecular Layer of the Dentate Gyrus (GC-ML-DG), Hippocampal Amygdala Transition Area (HATA). [file Table_3.pdf]

Table 3. Hippocampal subfiled abnormality in treatment naive MDD patients

	Control vs Baseline	
**Left Hippocampus**	**t**	**p**
Tail	0.3727	0.721
Subiculum	1.965	0.051
CA1	2.44	**0.016**
Fissure	0.597	0.551
Presubiculum	1.662	0.098
Parasubiculum	3.37	**<0.001**
Molecular layer	2.83	**0.005**
GC-ML-DG	3.21	**0.002**
CA3	4.76	**<0.001**
CA4	3.53	**<0.001**
Fimbria	0.738	0.461
HATA	4.54	**<0.001**
Whole	2.67	**0.008**
**Right Hippocampus**		
Tail	0.626	0.532
Subiculum	1.78	0.077
CA1	2.43	**0.016**
Fissure	-0.452	0.652
Presubiculum	1.022	0.308
Parasubiculum	3.74	**<0.001**
Molecular layer	3.06	**0.003**
GC-ML-DG	4.07	**<0.001**
CA3	5.49	**<0.001**
CA4	4.44	**<0.001**
Fimbria	2.99	**0.003**
HATA	2.67	**0.008**
Whole	2.962	**0.003**
