# Supplementary material for: Treatment-Specific Hippocampal Subfield Volume Changes With Antidepressant Medication or Cognitive-Behavior Therapy in Treatment-Naive Depression
Source: Front Psychiatry. 2021 Dec 24;12:718539. doi: 10.3389/fpsyt.2021.718539 (PMC8739262; doi:10.3389/fpsyt.2021.718539)
Supplement: Supplementary Table 4 — Baseline effect between Baseline and Week 12. Cornu Ammonis (CA), Granule Cell Molecular Layer of the Dentate Gyrus (GC-ML-DG), Hippocampal Amygdala Transition Area (HATA). [file Table_4.pdf]

Table 4. Hippocampal subfield volume changes regardless of treatment

	Baseline vs Week12	
**Left Hippocampus**	F	p
Tail	3.457	0.065
Subiculum	4.31	**0.039**
CA1	4.08	**0.045**
Fissure	0.274	0.602
Presubiculum	0.641	0.424
Parasubiculum	0.778	0.379
Molecular layer	2.704	0.102
GC-ML-DG	0.782	0.378
CA3	0.810	0.369
CA4	0.470	0.494
Fimbria	4.098	**0.044**
HATA	11.428	**<.001**
Whole	4.106	**0.044**
**Right Hippocampus**		
Tail	4.935	**0.028**
Subiculum	0.0483	0.826
CA1	0.248	0.619
Fissure	3.66	0.058
Presubiculum	0.0139	0.906
Parasubiculum	0.271	0.603
Molecular layer	0.0678	0.795
GC-ML-DG	0.0631	0.802
CA3	0.767	0.782
CA4	1.02E-04	0.992
Fimbria	0.108	0.743
HATA	5.67	**0.018**
Whole	0.538	0.464
