# Supplementary material for: Treatment-Specific Hippocampal Subfield Volume Changes With Antidepressant Medication or Cognitive-Behavior Therapy in Treatment-Naive Depression
Source: Front Psychiatry. 2021 Dec 24;12:718539. doi: 10.3389/fpsyt.2021.718539 (PMC8739262; doi:10.3389/fpsyt.2021.718539)
Supplement: Supplementary Table 5 — Chronicity effect between Baseline and Week 12. Cornu Ammonis (CA), Granule Cell Molecular Layer of the Dentate Gyrus (GC-ML-DG), Hippocampal Amygdala Transition Area (HATA). [file Table_5.pdf]

Table 5. Chronicity * Time Interaction

	Remitters	
**Left Hippocampus**	F	p
Tail	0.392	0.533
Subiculum	1.998	0.161
CA1	0.578	0.449
Fissure	0.206	0.651
Presubiculum	1.432	0.235
Parasubiculum	1.627	0.206
Molecular layer	1.486	0.226
GC-ML-DG	0.399	0.529
CA3	0.612	0.436
CA4	0.573	0.451
Fimbria	0.102	0.750
HATA	0.297	0.587
Whole	1.352	0.248
**Right Hippocampus**		
Tail	0.451	0.504
Subiculum	1.058	0.307
CA1	0.992	0.322
Fissure	2.929	0.091
Presubiculum	0.002	0.968
Parasubiculum	0.002	0.967
Molecular layer	1.080	0.302
GC-ML-DG	0.248	0.620
CA3	1.672	0.200
CA4	0.198	0.657
Fimbria	3.219	0.076
HATA	1.394	0.241
Whole	0.684	0.410
