# Supplementary material for: Treatment-Specific Hippocampal Subfield Volume Changes With Antidepressant Medication or Cognitive-Behavior Therapy in Treatment-Naive Depression
Source: Front Psychiatry. 2021 Dec 24;12:718539. doi: 10.3389/fpsyt.2021.718539 (PMC8739262; doi:10.3389/fpsyt.2021.718539)
Supplement: Supplementary Table 6 — Treatment effect between Baseline and Week 12. Cornu Ammonis (CA), Granule Cell Molecular Layer of the Dentate Gyrus (GC-ML-DG), Hippocampal Amygdala Transition Area (HATA). [file Table_6.pdf]

Table 6. Treatment-specific hippocampal subfield volume changes in AD-treated and CBT-treated remitters

	**Remitter**	
**Left Hippocampus**	F	p
Tail	0.307	0.581
Subiculum	0.333	0.565
CA1	1.118	0.293
Fissure	0.949	0.333
Presubiculum	0.149	0.700
Parasubiculum	0.031	0.861
Molecular layer	0.113	0.737
GC-ML-DG	0.058	0.811
CA3	0.314	0.577
CA4	0.050	0.824
Fimbria	0.134	0.715
HATA	3.070	0.083
Whole	0.329	0.568
**Right Hippocampus**		
Tail	4.611	**0.035**
Subiculum	0.960	0.330
CA1	2.267	0.136
Fissure	0.030	0.863
Presubiculum	2.316	0.132
Parasubiculum	0.393	0.393
Molecular layer	1.163	0.284
GC-ML-DG	0.818	0.368
CA3	2.116	0.149
CA4	0.310	0.579
Fimbria	0.119	0.119
HATA	4.857	**0.030**
Whole	3.509	0.0650
