# Supplementary material for: Treatment-Specific Hippocampal Subfield Volume Changes With Antidepressant Medication or Cognitive-Behavior Therapy in Treatment-Naive Depression
Source: Front Psychiatry. 2021 Dec 24;12:718539. doi: 10.3389/fpsyt.2021.718539 (PMC8739262; doi:10.3389/fpsyt.2021.718539)
Supplement: Supplementary Table 7 — Outcome effect between Baseline and Week 12. Cornu Ammonis (CA) Granule Cell Molecular Layer of the Dentate Gyrus (GC-ML-DG), Hippocampal Amygdala Transition Area (HATA). [file Table_7.pdf]

Table 7. Outcome * Time Interaction

	**All**		**Remitter + Nonresponder**		**AD (Remitters + Nonresponder)**		**CBT (Remitters + Nonresponders)**	
**Left Hippocampus**	F	p	F	p	F	p	F	p
Tail	2.333	0.076	4.847	**0.030**	6.806	**0.011**	0.078	0.782
Subiculum	0.216	0.885	2.66E-04	0.987	0.651	0.422	1.467	0.236
CA1	0.477	0.699	6.36E-03	0.937	0.189	0.665	0.333	0.568
Fissure	1.885	0.134	0.170	0.681	0.304	0.583	6.36E-04	0.980
Presubiculum	0.684	0.563	0.960	0.329	3.124	0.081	0.298	0.589
Parasubiculum	0.349	0.790	0.406	0.525	0.931	0.338	0.106	0.747
Molecular layer	0.306	0.821	0.529	0.469	1.437	0.234	0.194	0.663
GC-ML-DG	0.183	0.908	0.169	0.681	0.442	0.508	0.074	0.788
CA3	0.172	0.915	0.164	0.686	0.389	0.535	0.056	0.815
CA4	0.291	0.832	0.198	0.657	0.354	0.554	4.26E-03	0.948
Fimbria	0.277	0.842	0.328	0.568	0.711	0.402	0.382	0.542
HATA	0.448	0.719	0.322	0.572	0.918	0.341	0.744	0.396
Whole	0.326	0.806	0.763	0.384	1.921	0.170	0.207	0.653
**Right Hippocampus**								
Tail	0.385	0.764	0.885	0.349	6.373	**0.014**	1.650	0.210
Subiculum	0.325	0.807	0.053	0.819	1.239	0.269	1.318	0.261
CA1	0.899	0.443	0.112	0.739	0.840	0.362	1.267	0.270
Fissure	1.198	0.312	0.033	0.856	0.186	0.667	1.014	0.323
Presubiculum	0.796	0.498	0.634	0.428	3.465	0.066	1.010	0.324
Parasubiculum	0.148	0.931	0.208	0.649	0.187	0.667	4.10E-03	0.949
Molecular layer	1.144	0.333	0.837	0.362	2.260	0.137	0.525	0.475
GC-ML-DG	0.955	0.415	1.293	0.258	1.456	0.231	8.82E-03	0.926
CA3	0.770	0.512	0.602	0.439	0.626	0.431	3.43E-04	0.985
CA4	1.089	0.355	1.571	0.213	1.406	0.239	0.161	0.691
Fimbria	0.235	0.872	0.287	0.593	2.073	0.154	1.869	0.182
HATA	0.177	0.912	0.360	0.550	2.19E-03	0.963	2.837	0.103
Whole	0.829	0.480	0.792	0.375	2.931	0.091	10.385	0.317
