# Supplementary material for: Treatment-Specific Hippocampal Subfield Volume Changes With Antidepressant Medication or Cognitive-Behavior Therapy in Treatment-Naive Depression
Source: Front Psychiatry. 2021 Dec 24;12:718539. doi: 10.3389/fpsyt.2021.718539 (PMC8739262; doi:10.3389/fpsyt.2021.718539)
Supplement: Supplementary Table 8 — HDRS score changes with volume change. Cornu Ammonis (CA), Granule Cell Molecular Layer of the Dentate Gyrus (GC-ML-DG), Hippocampal Amygdala Transition Area (HATA), Hamilton Depression Rating Scale (HDRS). [file Table_8.pdf]

Table 8. Hippocampal subfield volume change associated with clinical outcome (% change of HDRS-17)

	All	
**Left Hippocampus**	F	p
Tail	2.841	0.094
Subiculum	0.043	0.836
CA1	3.470E-04	0.985
Fissure	0.225	0.636
Presubiculum	0.257	0.613
Parasubiculum	0.411	0.522
Molecular layer	0.651	0.421
GC-ML-DG	0.361	0.549
CA3	0.120	0.730
CA4	0.516	0.473
Fimbria	0.118	0.732
HATA	0.0853	0.771
Whole	0.563	0.454
**Right Hippocampus**		
Tail	0.306	0.581
Subiculum	0.029	0.864
CA1	0.594	0.442
Fissure	1.150E-04	0.991
Presubiculum	0.969	0.326
Parasubiculum	0.103	0.749
Molecular layer	1.153	0.285
GC-ML-DG	1.503	0.222
CA3	0.337	0.562
CA4	1.343	0.248
Fimbria	4.53E-03	0.946
HATA	1.236	0.268
Whole	0.787	0.376
